# Incidence and predictor of diabetic foot ulcer and its association with change in fasting blood sugar among diabetes mellitus patients at referral hospitals in Northwest Ethiopia, 2021

**DOI:** 10.1371/journal.pone.0274754

**Published:** 2022-10-13

**Authors:** Habtamu Wagnew Abuhay, Melaku Kindie Yenit, Haileab Fekadu Wolde

**Affiliations:** 1 Epidemiology Program, College of Medicine and Health Sciences, University of Gondar, Gondar, Ethiopia; 2 Department of Epidemiology and Biostatistics, Institute of Public Health, College of Medicine and Health Sciences, University of Gondar, Gondar, Ethiopia; GSVM Medical College, INDIA

## Abstract

**Background:**

Diabetes mellitus is one of the global public health problems and fasting blood sugar is an important indicator of diabetes management. Uncontrolled diabetes can lead to diabetic foot ulcers, which is a common and disabling complication. The association between fasting blood glucose level and the incidence of diabetic foot ulcers is rarely considered, and knowing its predictors is good for clinical decision-making. Therefore, the aim of this study was to determine the incidence and predictors of diabetic foot ulcers and its association with changes in fasting blood sugar among diabetes mellitus patients at referral hospitals in Northwest Ethiopia.

**Methods:**

A multicenter retrospective follow-up study was conducted at a referral hospital in Northwest Ethiopia. A total of 539 newly diagnosed DM patients who had follow-up from 2010 to 2020 were selected using a computer-generated simple random sampling technique. Data was entered using Epi-Data 4.6 and analyzed in R software version 4.1. A Cox proportional hazard with a linear mixed effect model was jointly modeled and 95% Cl was used to select significant variables. AIC and BIC were used for model comparison.

**Result:**

A total of 539 diabetes patients were followed for a total of 28727.53 person-month observations. Overall, 65 (12.1%) patients developed diabetic foot ulcers with incidence rate of 2.26/1000-person month observation with a 95% CI of [1.77, 2.88]. Being rural (AHR = 2.30, 95%CI: [1.23, 4.29]), being a DM patient with Diabetic Neuropathy (AHR = 2.61, 95%CI: [1.12, 6.06]), and having peripheral arterial disease(PAD) (AHR = 2.96, 95%CI: [1.37, 6.40]) were significant predictors of DFU. The time-dependent lagged value of fasting blood sugar change was significantly associated to the incident of DFU (α = 1.85, AHR = 6.35, 95%CI [2.40, 16.79]).

**Conclusion and recommendation:**

In this study, the incidence of DFU was higher than in previous studies and was influenced by multiple factors like rural residence, having neuropathy, and PAD were significant predictors of the incidence of DFU. In addition, longitudinal changes in fasting blood sugar were associated with an increased risk of DFU. Health professionals and DM patients should give greater attention to the identified risk factors for DFU were recommended.

## Introduction

Diabetes mellitus (DM) is a chronic metabolic disease characterized by an elevated level of blood glucose or blood sugar. It is one of the global public health problems [1]. In 2019, about 463 million people have been affected by diabetes. The African region is estimated to have 19.4 million adults living with diabetes [2]. Ethiopia is one of the top five African countries with the highest prevalence of diabetes mellitus, with around 1.9 million diabetic patients [3]. When diabetes mellitus is not properly managed, it can lead to various macro or microvascular complications, such as diabetic neuropathy, nephropathy, peripheral arterial diseases, stroke, and diabetic foot ulcers [4, 5].

Diabetes foot ulcers are the most common type of diabetes complication. It is an ulceration of the foot that subsequently leads to amputation of the leg [6]. Pooled estimates on the global prevalence of diabetic foot ulcers among diabetes mellitus patients were 6.3%, and in Africa, 7.2% of diabetes patients have diabetes foot ulcers, with Ethiopia having the highest incidence of DFU at 17.05% [7, 8]. As a result of their prolonged hospitalization, patients with DFU fail to heal completely and are prone to infection, tissue necrosis, and gangrene [9, 10]. Consequently, it causes lower limb amputations and poor health-related quality of life [11].

Despite the incidence of diabetes, foot ulcers are affected by various factors; socio-demographic variables, duration of diabetes, uncontrolled diabetes, and co-morbidities are the most reported risk factors [7, 12–16]. Fasting blood sugar (FBS) is an important clinical biomarker for diabetes diagnosis and management that helps to track disease progress and prevent diabetes complications [17]. Studies also show that FBS level is also highly correlated with DFU, and optimal FBS control can reduce the occurrence of lower limb amputations [18].

In Ethiopia, there are few survival studies on the incidence of DFU among DM patients, but to the best of my knowledge, there are no studies that assess the association between longitudinal biomarkers of FBS with the incidence of DFU. Therefore, this study aimed to assess the incidence and predictors of diabetic foot ulcers and its association with changes in fasting blood sugar among DM patients.

## Methods

### Study design and setting

An institution-based retrospective follow-up study was conducted from January 1, 2010 to December 30, 2020 G.C.

The study was conducted at the University of Gondar compressive specialized hospital, Felege Hiwot referral hospital, and Debre Tabor referral hospital, which are located in the Northwest Ethiopia, Amhara National Regional State.

### Sample and population

All DM patients who had follow-up at referral hospitals in Northwest Ethiopia were the source population.

All newly diagnosed diabetes patients who had follow-up at the University of Gondar compressive specialized hospital, Felege Hiwot referral hospital, and Debre Tabor referral hospitals from January 1, 2010 to August 2020, and whose age was greater than or equal to 15, were the study participants.

A total of 539 study subjects was estimated. For the survival objective sample, size was determined using predictors significantly associated with time to DFU from previous studies done in Ghana [8, 19]. Using the following assumptions, Power = 90% ᾳ = 0.01, ß = 0.1 withdrawal = 0.1 then adding 10% incompleteness and considering design effect 1.5. and the Schoenfeld formula was used to determine the sample size [20].

DM Patients whose dates of registration and diagnosis of a diabetic foot ulcer were unknown, as well as patients who had already developed a diabetic foot ulcer at the time the study began, were excluded.

In the Amhara region, there are eight referral hospitals, and the study was conducted in three randomly selected referral hospitals. After random selection, we distributed the sample size proportionally among those hospitals. Study participants charts were selected by using computer-generated simple random sampling method. There are 4700 newly diagnosed DM patients in the selected referral hospitals, with 245 of the 2136 DM patients at the University of Gondar Compressive specialized hospital, 189 of the 1647 DM patients at Felege Hiwot referral hospital, and 105 of the 917 DM patients at Debre Tabor referral hospital were selected. A sampling frame was prepared by collecting the identification numbers of DM patients from the registration book. Finally, participant charts for the study were selected by using computer-generated simple random sampling method.

### Data collection procedure and measurement

A structured English version data collection checklist was developed based on existing DM patients’ medical records and other previous similar studies. The check list had three parts. The first part is about socio-demographic characteristics; the second is for clinical and physiologic characteristics; and the last part is for recording the time-dependent covariates (fasting blood sugar level).

The dependent variable for this study was diabetic foot ulcer, and it was defined as non-traumatic lesions of the skin (partial or full thickness) on the foot of a person who has diabetes mellitus.

The independent variables were socio-demographic variables: age, sex, and residence.

Physiologic factors: Protein urea, High-density lipoprotein (mg/dl), Low-density lipoprotein (mg/dl), Triglyceride (mg/dl), Creatinine (mg/dl), Total cholesterol (mg/dl), Systolic blood pressure (mmHG), diastolic blood pressure(mmHG).

Clinical factors: BMI, type of treatment, family history of DM, Co-morbidity (hypertension), duration of DM, type of DM, diabetic neuropathy, diabetic retinopathy, diabetic nephropathy, diabetic nephropathy, peripheral arterial disease, stroke. Time varying factor: Repeatedly measured FBS in mg/dl.

A Structured and pre-tested checklist was used to collect the data. Twelve-BSc nurses as data collectors reviewed and extracted the data from patient charts and registries.

### Data processing and statistical analysis

Data was entered into EpiData version 4.4 and analyzed by R version 4.1 software. Before analysis, data was edited, verified, cleaned, coded, and merged as necessary to make it suitable for analysis. Descriptive statistics for categorical variables were done. Continuous variables were also described. Individual profile plots were constructed to provide a rough image of how subjects evolved and to provide explanations for variation between and within subjects. In addition, average profile plots were constructed.

Then model comparison was done for the longitudinal sub-model by using AIC and BIC. In addition, the normality assumption for FBS was checked for the linear mixed effect model. Survival part: incidence of diabetic foot ulcer was estimated using Kaplan-Meier (KM) and Log-rank test to compare survival time between groups of categorical variables. Proportional hazard assumptions (PHA) were checked before fitting the survival sub model by the cumulative log hazard plot and the Schoenfeld residuals method.

The factors significantly associated with DFU in the bi-variable analysis at p-values less than 0.2 were included in the multivariable survival model and further examined in the joint model. The best fitting survival and longitudinal sub models were selected based on AIC. Then, data was first analyzed using a linear mixed effect model for longitudinal sub model and best fitted survival models separately.

The best fitting survival and longitudinal sub models were selected and further examined in the joint model. (Cox proportional hazard with a linear mixed effect model jointly modeled). Then joint models were fitted with different parameterizations using the JM package of R software.

The best parameterization for our data was selected using AIC and BIC from different parameterization of joint models. The association parameter (alpha value) from fitted joint model was used to assess the association between longitudinal FBS change and Diabetic foot ulcer. In a multivariable joint model, a covariate with p-value <0.05 that contained in the 95% confidence interval was considered significant.

### Ethical approval and consent to participate

Ethical approval was obtained from the ethical review board of the University of Gondar, College of Medicine and Health Science, Institute of Public Health prior to enrolment. Before we accessed the data from medical records, it was fully anonymized, and the IRB waived the requirement for informed consent. In addition, official permission was obtained from the selected referral hospitals’ clinical director. Privacy of the patients’ medical records was maintained, names were not included, and the checklist was kept locked.

## Results

### Socio-demographic and clinical characteristics

In this study, a total of 539 newly diagnosed DM patients were included in the study. The mean (SD) age of study participants was 46.05 (16.02) years. The majority of study participants 301 (55.8%) and 287 (53.3%) were males and urban residents, respectively. Among the study participants nearly two-third of them, 345 (64.0%) and 105 (20.2%), respectively, were TIIDM and had a family history of diabetes. More than half 272 (50.5) of patients were on oral hypoglycemic agent (OHA) treatment, which was followed by insulin treatment 221 (41.0%). Seventy-eight (15.9%) of the study participants had positive proteinuria at baseline (Table 1).

**Table 1:** Socio-demographic and clinical characteristics of DM patients on treatment at UoGCSH, FRH, DTRH in Northwest Ethiopia, 2010 to 2020.

Variables	Categories	Frequency	Percent (%)
**Sex**	Male	301	55.8
	Female	238	44.2
**Residence**	Urban	287	53.3
	Rural	252	46.7
**Family History of DM**	Yes	105	20.2
	No	349	63.4
	Unknown	85	16.4
**Type of DM**	TIDM	194	36.0
	TIIDM	345	64.0
**Type of treatment**	OHA	272	50.5
	Insulin	221	41.0
	Both	46	8.5
**Duration of DM**	< 5 year	397	73.6
	≥ 5 year	141	26.4
**Hypertension**	Yes	162	30.3
	No	373	69.7
**Proteinuria**	Positive	78	15.9
	Negative	413	84.1
**Last status of patient**	Alive	408	75.7
	Lost follow-up	58	10.7
	Died	8	1.5
	Event	65	12.1
**Category of hospital**	FHRH	173	32.1
	DTRH	76	14.1
	UoGCSH	290	53.8

TIDM: Type one diabetic mellitus, TIIDM: Type two diabetic mellitus, OHA: Oral hypoglycemic agent, FHRH: Felege Hiwot referral hospital, DTRH: Debre Tabor referral hospital, UoGCSH: University of Gondar referral hospital.

### Baseline physiologic characteristics and diabetic complication

Twenty-six (11.9%) and six (2.7%) of the patients had high triglyceride and total cholesterol levels, respectively. More than half of the patients (59.3%) had an HDL-C level greater than 40mg/dl and 45 (19.5%) of the study participants had an LDL-C level greater than 100mg/dl. Eighteen (18.8%) of patients had a BMI greater than or equal to 25kg/m^2^. Regarding diabetic related complications, 76 (14.1%) and 41 (7.6%) had diabetic retinopathy and diabetic Neuropathy respectively (Table 2).

**Table 2:** Baseline physiologic characteristics and diabetic-related complication among DM patients on treatment at UoGCSH, FHRH, and DTRH in Northwest Ethiopia, 2010 to 2020.

Variables	Categories	Frequency	Percent (%)
**Total cholesterol(mg/dl)**	<200	187	83.1
	200–239	32	14.2
	≥240	6	2.7
**Triglyceride(md/dl)**	<150	150	69.2
	150–199	41	18.9
	≥200	26	11.9
**LDL-C(md/dl)**	<100	186	80.5
	≥100	45	19.5
**HDL-C(md/dl)**	<40	92	40.7
	≥40	134	59.3
**BMI**	<18.5	1	1.0
	18.5–24.9	77	80.2
	≥25	18	18.8
**SBP(mmHG)**	<140	379	70.3
	≥140	160	29.7
**DBP (mmHG)**	<90	471	87.4
	≥90	68	12.6
**Retinopathy**	No	463	85.9
	Yes	76	14.1
**Neuropathy**	No	498	92.4
	Yes	41	7.6
**Nephropathy**	No	501	92.9
	Yes	38	7.1
**Stroke**	no	517	95.9
	Yes	22	4.1
**CHD**	No	511	94.8
	Yes	28	5.2
**Peripheral arterial disease**	no	515	95.6
	Yes	24	4.4

md/dl: Milligram per deciliter, LDL-C: Low-density lipoprotein creatinine, HDL-C: High-density lipoprotein creatinine, BMI: Body mass index, SBP: Systolic blood pressure, DBP: Diastolic blood pressure, mmHG: Millimetre of mercury, CHD: Chronic heart disease.

### Incidence of diabetic foot ulcer

**Fig 1:**
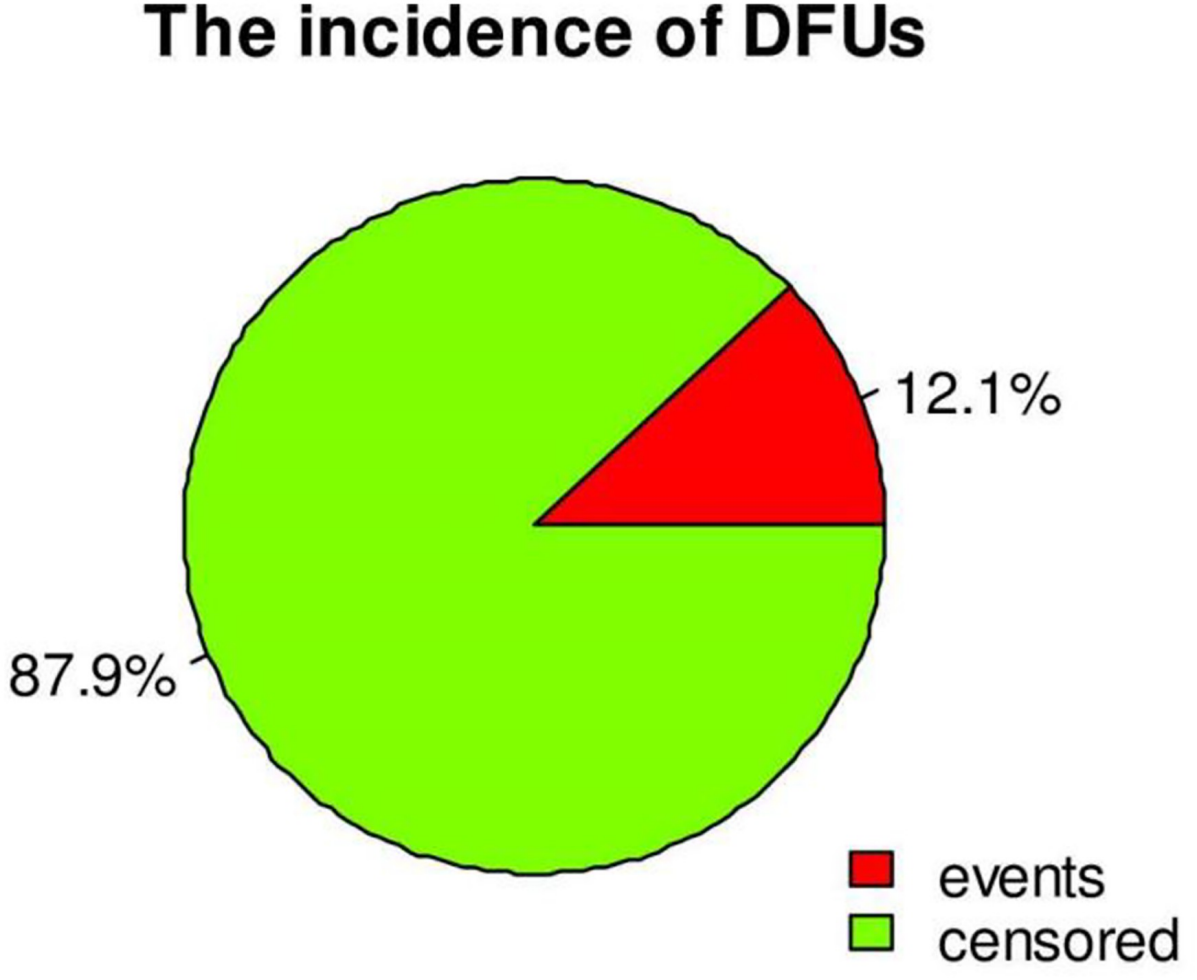
Incidence of DFU among DM patients on treatment at Northwest Ethiopia, from 2010 to 2020.

The patients were followed for a minimum of 6.3 and a maximum of 120 months, with a median follow-up time of 64 months [IQR, [43.9, 85.5]. Out of 539 study participants followed, a total of 65 (12.1%), 95% CI [9.49–15.18] developed DFU in 28727.53 person months (PM) of observations. The incidence density was 2.26/1000 PM with 95% CI of [1.77, 2.88]. Or 2.71 per 100 PY 95% CI of [2.12, 3.46] (Fig 1).

### Predictors for the incidence of DFU

The joint modeling gives associated factors for both changes in FBS and the incidence of diabetic foot ulcers. The result showed that the 3 month lagged value of fasting blood sugar was significantly associated with the risk of DFU. Table 3; revealed that strong association between the longitudinal bio-marker (log of FBS) and the risk of DFUs (α = 1.84, p-value 0.000), meaning that a unit increase in the past 3-month value of log of fasting blood sugar level corresponds to an exp (1.84) = 6.3 fold increase in the todays risk of DFU.

**Table 3:** Joint model (Survival sub models with time dependent association parameters) for DM patients on treatment in Northwest Ethiopia, 2010 to 2020.

Variables	Categories	Diabetic foot ulcer		AHR [95% Cl]	P-value
		Censored	Event		
**Sex**	Male	257	44	1	
	Female	217	21	0.77 [0.42, 1.38]	0.387
**Residence**	Urban	268	19	1	
	Rural	206	46	**2.30 [1.23, 4.29]**	**0.008** ***
**Hypertension**	No	341	36	1	
	Yes	133	29	1.10 [0.56, 2.14]	0.770
**Family history of DM**	No	383	51	1	
	Yes	91	14	0.59 [0.29, 1.20]	0.148
**Protein Urea**	Negative	416	45	1	
	Positive	55	23	1.24 [0.65, 2.35]	0.499
**Diabetic complication**	No	397	17	1	
	Yes	77	48	1.59 [0.65, 3.90]	0.302
**Retinopathy**	No	449	45	1	
	Yes	25	20	2.05 [0.98, 4.26]	0.053
**Neuropathy**	No	463	35	1	
	Yes	11	30	**2.61 [1.12, 6.06]**	**0.025** **
**Nephropathy**	No	450	51	1	
	Yes	24	14	2.17 [0.99, 4.78]	0.052
**PAD**	No	465	50	1	
	Yes	9	15	**2.96 [1.37, 6.40]**	**0.005** ***
Association parameter (lag of 3 month)					
**Association parameter (lag = 3 month)**				**6.35 [2.40, 16.79]**	**0.000** ***

***expressed as p-value <0.001,

** p-value <0.01,

* p-value 0.05,

DFU: Diabetic foot ulcer; PAD: Peripheral arterial disease, OHA: Oral hypoglycemic agent.

In addition, residence, neuropathy, and peripheral arterial disease were significant predictors of incident of diabetic foot ulcers. After adjusting for other variables in the model, the hazard of developing DFU is 2.3 times higher among rural patients as compared to urban patients (AHR = 2.3, 95%CI: [1.23, 4.29]). The hazard of developing DFU is 2.61 times higher among DM patients with neuropathy as compared to their counterparts, keeping other variables constant (AHR = 2.6, 95%CI: [1.12, 6.06]). After adjusting for other variables in the model, the hazard of experiencing DFU was 2.9 times higher for DM patients with PAD as compared to their counter parts (AHR = 2.9, 95%CI: [1.37, 6.40]) as presented in (Table 3).

## Discussion

Diabetic foot ulcer is a major global public health concern and an important cause of lower limb amputation, hospitalization, morbidity, and mortality. Therefore, this study investigated the incidence and predictors of diabetic foot ulcers as well as the effect of fasting blood sugar change on the risk of DFUs among newly diagnosed DM patients on treatment in Northwest Ethiopia.

In this study, factors like residence, diabetic neuropathy, and peripheral arterial disease were also found to be significantly associated with the risk of DFU. In addition, there was a strong association between longitudinal measurement of FBS (log of FBS) and the risk of developing diabetic foot ulcer.

In this study, 12.1% of the study participants had DFU with the incidence density of 2.26/1000 PM or 2.71 per 100 PY of observation. This result is in line with previous studies in Ethiopia where the pooled estimate of DFU was 12.9% [21]. However this finding is lower than that of a study in Nekemte, Ethiopia were 17.8% and incidence of 4 per 100 PY of observation developed DFU in Felege hiwot referral hospital, Ethiopia, [8, 22]. The possible reason for this discrepancy might be due to a difference in the follow-up period for the studies and recruitment criteria.

However, this study revealed a higher incidence of DFU than studies done in both Europe and Asia, where the pooled estimates of diabetic foot ulcers were 5.1% and 5.5%, respectively [7]. In Iran on the cumulative incidence of diabetic foot ulcers was 5.6% [23]. A study in United Kingdom found an overall incidence of 4.4% [24]. Possible reasons for these differences may be due to differences in lifestyle status, socio-economic and socio-cultural variations of study participants.

According to our findings, the risk of DFU is much higher in DM patients who live in rural areas than in DM patients who live in urban areas. This result is in agreement with previous studies in Ethiopia and India [12, 25]. The possible reasons could be due to being rural and having poor health care seeking behavior [26], low income and educational status [27]. DM patients who live in urban areas and earn a high income were significant determinants of good self-care practice [27]. Patients who had not practiced foot self-care were likely to develop a diabetic foot ulcer. In addition, DM patients in rural areas were less knowledgeable than in urban areas [28]. Therefore, less knowledge may lead to inadequate blood glucose control and prone to diabetic foot ulcers.

According to our findings, the hazard of experiencing DFU was higher among DM patients with diabetic neuropathy as compared to patients who did not have neuropathy. This result is in agreement with previous studies conducted in Southwest Ethiopia [28], India [25], and Jordan [29]. This could be due to loss of sensation, which is typically seen in patient with diabetes neuropathy that affects the sensory nerves responsible for detecting sensations such as temperature or pain. Then, after it causes local paresthesia, or lack of sensation, pressure points on the foot lead to micro trauma, breakdown of overlying tissue, and eventually lead to foot ulcerations.

In our study, it was found that the DM patients who had peripheral arterial disease had 2.96 increases in the risk of developing DFU as compared to those DM patients without peripheral arterial disease. This result was similar to other studies done in Peru [23]. Patients who had peripheral arterial disease three times increased the risk of developing DFU. This could be due to patients with PAD having narrowed blood vessels and reducing blood flow to the legs or lower extremities. This decreased blood flow lead to ischemia, which can cause nerve and other tissue damage that predispose DM patients to DFU.

From the results of the current study, we observed that the lag of the current level of the longitudinal FBS was strongly associated with the risk of diabetic foot ulcer. For patients with the same covariates in the model at baseline and who have the same underlying level of FBS at time *t* − 3, increasing FBS by 50% increases the risk of DFU by 6.35-fold.

Even though no study was done to assess the association of longitudinal FBS change with the risk of DFU among DM patients, but there are studies that shows DFU is highly related to blood glucose levels among DM patients. According to studies conducted in the United States, the incidence of diabetic foot ulcers is related to an elevated glycemic level [30]. In addition, a study conducted in Iran showed that poor blood glucose control associated with diabetic foot ulcers [31]. This might be due to increased fasting blood glucose, indicating poor blood glucose control and might relate to diabetic foot ulcer.

The clinical importance of the findings of this is study was to provide information for health professionals and patients as high FBS can be used as a signal to screen DM patients for DFU and early detection of DFU and its complications. In addition, it provides information about factors that are associated with the risk of diabetic foot ulcers. Therefore, it helps to minimize the risk and maximize effort on prevention of having the problem. The public health importance of this study is to prevent disability, economic loss, and loss of productivity associated with DFU by identifying the variable most significantly associated with DFU.

## Conclusion

In the current study, the incidence of DFU was relatively higher compared with a previous similar study in Ethiopia. Fasting blood sugar changes were associated with an increased risk of DFU. Besides, rural residence, having neuropathy, and peripheral arterial disease were significant predictors of incidence of DFU. Therefore, close monitoring of the change in fasting blood sugar for patients with diabetic mellitus and diabetic patients who live in rural residence, as well as those who have diabetic neuropathy and peripheral arterial disease, should be considered to reduce DFU.

## Recommendations

Based on the findings of the current study, the following recommendations are made for the concerned bodies. Health professionals should give greater attention to DM patients with the identified risk factors for DFU. Patients with diabetes mellitus and neuropathy or peripheral arterial disease should carefully monitor and control their blood glucose levels. Further studies on the topic by including behavioral factors like alcohol consumption history and primary data with prospective studies are recommended.

## Abbreviations

AIC: Akaike’s Information Criteria
AOR: Adjusted Odds Ratio
BIC: Bayesian Information Criteria
BP: Blood Pressure
CDC: Center for Disease Control
CI: Confidence Interval
DBP: Diastolic Blood Pressure
DFU: Diabetic Foot Ulcer
DM: Diabetes Mellitus
DTRH: Debre Tabor Referral Hospital
FBS: Fasting Blood Sugar
FHRH: Felege Hiwot Referral Hospital
HDL: High-Density Lipoprotein
HR: Hazard Ratio
IRB: Ethical Review Board
KM: Kaplan Meier
LDL: Low-Density Lipoprotein
OHA: Oral Hypoglycemic Agents
PAD: Peripheral Arterial Disease
PHA: Proportional Hazard Assumption
PI: Principal Investigator
PY: Person Years
SBP: Systolic Blood Pressure
SD: Standard Deviation
UoG: University of Gondar
UoGCSH: University of Gondar Compressive Specialized Hospital
WHO: World Health Organization
